# The histone deacetylase inhibitor nicotinamide exacerbates neurodegeneration in the lactacystin rat model of Parkinson's disease

**DOI:** 10.1111/jnc.14599

**Published:** 2018-11-26

**Authors:** Ian F. Harrison, Nicholas M. Powell, David T. Dexter

**Affiliations:** ^1^ UCL Centre for Advanced Biomedical Imaging Division of Medicine University College London London UK; ^2^ Parkinson's Disease Research Group Division of Brain Sciences Department of Medicine Centre for Neuroinflammation and Neurodegeneration Imperial College London London UK; ^3^ Translational Imaging Group Centre for Medical Image Computing University College London London UK

**Keywords:** histone deacetylase inhibitor, lactacystin, neurodegeneration, nicotinamide, Parkinson's disease, sirtuins

## Abstract

Histone hypoacetylation is associated with dopaminergic neurodegeneration in Parkinson's disease (PD), because of an imbalance in the activities of the enzymes responsible for histone (de)acetylation. Correction of this imbalance, with histone deacetylase (HDAC) inhibiting agents, could be neuroprotective. We therefore hypothesize that nicotinamide, being a selective inhibitor of HDAC class III as well as having modulatory effects on mitochondrial energy metabolism, would be neuroprotective in the lactacystin rat model of PD, which recapitulates the formation of neurotoxic accumulation of altered proteins within the substantia nigra to cause progressive dopaminergic cell death. Rats received nicotinamide for 28 days, starting 7 days after unilateral injection of the irreversible proteasome inhibitor, lactacystin, into the substantia nigra. Longitudinal motor behavioural testing and structural magnetic resonance imaging were used to track changes in this model of PD, and assessment of nigrostriatal integrity, histone acetylation and brain gene expression changes post‐mortem used to quantify nicotinamide‐induced neuroprotection. Counterintuitively, nicotinamide dose‐dependently exacerbated neurodegeneration of dopaminergic neurons, behavioural deficits and structural brain changes in the lactacystin‐lesioned rat. Nicotinamide treatment induced histone hyperacetylation and over‐expression of numerous neurotrophic and anti‐apoptotic factors in the brain, yet failed to result in neuroprotection, rather exacerbated dopaminergic pathology. These findings highlight the importance of inhibitor specificity within HDAC isoforms for therapeutic efficacy in PD, demonstrating the contrasting effects of HDAC class III inhibition upon cell survival in this animal model of the disease.

**Open science badges:**

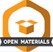

This article has received a badge for *Open Materials* because it provided all relevant information to reproduce the study in the manuscript. The complete Open Science Disclosure form for this article can be found at the end of the article. More information about the Open Practices badges can be found at https://cos.io/our-services/open-science-badges/.

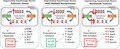

**Abbreviations used**
anova: analysis of variance
AOI: area of interest
asf: area sampling fraction
AUC: area under curve
BBB: blood–brain barrier
BDNF: brain‐derived neurotrophic factor
GDNF: glial‐derived neurotrophic factor
HDAC: histone deacetylase
HDACI: histone deacetylase inhibitor
hsf: height sampling fraction
i.p.: intraperitoneally
MPTP: 1‐methyl‐4‐phenyl‐1,2,3,6‐tetrahydropyridine
MRI: magnetic resonance imaging
MR: magnetic resonance
NAD+: nicotinamide adenine dinucleotide
PBS: phosphate‐buffered saline
PD: Parkinson's disease
PGC1α: peroxisome proliferator‐activated receptor gamma coactivator 1α
SEM: standard error or mean
SNpc: Substantia Nigra pars compacta
ssf: section sampling fraction
TBM: tensor‐based morphometry
TBS: tris‐buffered saline
TBS‐T: tris‐buffered saline tween
TH: tyrosine hydroxylase
αSyn: α‐synuclein

Parkinson's disease (PD) is the most common movement disorder, presenting clinically as a triad of symptoms, notably tremor, muscle rigidity and bradykinesia, as a result of neurodegeneration of the dopamine producing cells of the Substantia Nigra pars compacta (SNpc) in the brain (Dexter and Jenner 2013). The hallmarks of these degenerating neurons in PD are intracytoplasmic inclusions, Lewy bodies, containing altered proteins such as the synaptic protein, α‐synuclein (αSyn), which are thought to attribute to the nigrostriatal neurodegeneration observed in the disease (Dexter and Jenner 2013). It has been previously demonstrated that nuclear αSyn ‘masks’ the histone proteins around which DNA is coiled, preventing their acetylation and as such the resulting histone hypoacetylation is thought to contribute to neurodegeneration in PD (Kontopoulos *et al*. 2006). In addition, it has been observed that in neurodegenerative states, there is a critical imbalance between the activities of the two enzymes which control histone acetylation: histone acetyltransferases and histone deacetylases (HDACs), in favour of histone deacetylation, exacerbating the hypoacetylation of histone proteins (Rouaux *et al*. 2003). It has been hypothesized then that perhaps HDAC inhibitors (HDACIs) may act therapeutically by rectifying the misbalance between histone (de)acetylation, reducing histone hypoacetylation‐mediated neurodegeneration in PD (Harrison and Dexter 2013).

To date, four main classes of HDACs have been described: classes I (HDACs 1, 2, 3 and 8), IIa (HDACs 4, 5, 7 and 9), IIb (HDACs 6 and 10), III (Sirtuins 1–7) and IV (HDAC 11) (Xu *et al*. 2007), with numerous isoform selective and isoform non‐selective HDACIs now available. Appropriately, inhibitors of a number of these classes and sub‐classes of HDACs have shown to produce a neuroprotective effect in cell and animal models of PD (for reviews see Harrison and Dexter 2013; Sharma and Taliyan 2015). For example we have demonstrated previously that delayed systemic treatment with valproate, an inhibitor of HDAC classes I and IIa, causes neuroprotection and neurorestoration of dopaminergic neurons within the SNpc and ventral tegmental area in the lactacystin rat model of PD (Harrison *et al*. 2015, 2016). HDAC Class III, the sirtuins, however, are enzymatically and structurally disparate from the remaining three classes of ‘classical’ HDACs. And as such inhibitors of this class are relatively under investigated because of the unavailability of selective sirtuin inhibitors. Recent advances in the field, however, have highlighted sirtuin1 and 2 as potential targets in PD (Garske *et al*. 2007; Outeiro *et al*. 2007; Donmez and Outeiro 2013; Chen *et al*. 2015; Harrison *et al*. 2018), and as such recent years have witnessed the development of novel inhibitors of these HDACs for the study of their therapeutic benefit in neurodegeneration (Cui *et al*. 2014; Tatum *et al*. 2014; Di Fruscia *et al*. 2015; Sundriyal *et al*. 2017). At present, however, research is hampered by the lack of pharmacokinetic study of these novel agents, more specifically whether or not they are able to penetrate the blood–brain barrier efficiently enough to produce selective HDAC inhibition in the brain.

Nicotinamide (niacinamide or nicotinic amide), is an amide converted, *in vivo*, from its dietary precursor niacin (nicotinic acid, a.k.a. vitamin B3). This B vitamin is found in various food sources, most abundantly in beef, chicken, pork, fish, peanuts, mushrooms, green beans, sunflower seeds and avocado. Pharmacologically, nicotinamide does not have the same adverse effects as high doses of niacin (cutaneous flushing and itching) which occur incidental to niacin's conversion. Rather, it displays a linear relationship between maximum recorded plasma concentrations and the dose in grams in man, exhibits maximal plasma levels around 30 min after dosing, and higher doses maintain high plasma levels for up to 4 h (Dragovic *et al*. 1995). Being a precursor for nicotinamide adenine dinucleotide (NAD+), nicotinamide is known to non‐selectively inhibit HDAC class III through competition binding to the sirtuin HDAC's NAD+ binding site (Avalos *et al*. 2005), and given its relatively low molecular weight (122.12 g/mol) is known to easily cross the blood–brain barrier (Spector 1987). It has therefore gained increased interest as a neuroprotective agent in neurodegenerative conditions. Furthermore, nicotinamide is known to have modulatory effects on cellular energy metabolism, which is known to become defective in dopaminergic neurons in PD, and hence even greater focus has been placed upon the potential use of this agent in PD (Beal 2003).

In the absence of brain penetrant highly selective/potent sirtuin inhibitors, it is vitally important to assess whether non‐selective sirtuin inhibitors like nictotinamide are neuroprotective, particularly since it may also correct the cellular energy imbalance observed in PD. Importantly, this study therefore aims for the first time to investigate the neuroprotective effect of nicotinamide in an animal model which recapitulates the formation of neurotoxic accumulation of altered proteins within the SNpc to cause progressive dopaminergic cell death: the lactacystin rat model, and hence mimics closely the pathological process of PD. Furthermore, a delayed start study design will be used to model the clinical scenario in which a neuroprotective drug would be administered to an already degenerating system, hence adding novelty since this will replicate more accurately the drug effect in the early stages of PD. In addition, molecular and cellular analyses of study samples will attempt to elucidate the neuroprotective mechanism of nicotinamide's HDAC inhibition in this model.

## Methods

### Experimental animals

Animal procedures were previously approved by Imperial College London's Animal Welfare and Ethical Review Board and carried out according to the UK's Home Office Animals (Scientific Procedures) Act of 1986 (PPL No.: 70/7398). This study was not pre‐registered. A total of 33 adult male Sprague–Dawley rats (250 ± 10 g; Charles River, Margate, Kent, UK, RRID:MGI:5651135) were used in this study, transported to Imperial College London's animal holding facility 2 weeks prior to use, where they were group housed (2–3 per cage) in temperature (21 ± 1°C), and humidity (55 ± 10%) controlled individually ventilated cages, on a 12‐h (7 am to 7 pm) light–dark cycle. *Ad libitum* drinking water and rat chow were available for the duration of the study, which was supplement post‐surgery with wet rat diet for 7 days. ARRIVE guidelines (Kilkenny *et al*. 2010) were adhered to in reporting of experimental findings.

### Animal treatment groups

**Figure 1:**
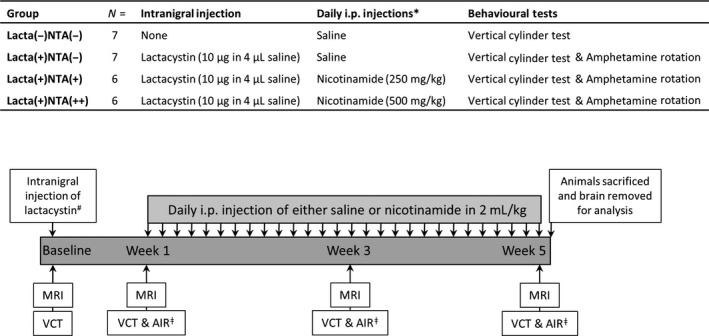
Animal treatment groups and study design. *All daily i.p. injections given as 2 mL/kg: saline injections given as 2 mL/kg empty saline; 500 mg/kg nicotinamide injections given as 2 mL/kg of 250 mg/mL solution of nicotinamide in saline; 250 mg/kg nicotinamide injections given as 2 mL/kg of 125 mg/mL solution of nicotinamide in saline. ^#^Only groups Lacta(+)NTA(−), Lacta(+)NTA(+) and Lacta(+)NTA(++) intranigrally injected with lactacystin. Control group remained surgically naïve. ^ǂ^Only groups lesioned with lactacystin were tested using the amphetamine‐induced rotation test at these time points. *N* numbers determined empirically based on previous work with the lactacystin rat model and magnitude of neuroprotection expected. VCT, vertical cylinder test; AIR, amphetamine‐induced rotations; MRI, magnetic resonance imaging.

Assessments of longitudinal changes in brain structure by Magnetic Resonance Imaging (MRI) and motor behavioural performance were made in four groups of animals (Fig. 1, *n* = 6–7, no sample size differences between the start and end of experiments). These measurements were taken at baseline, prior to lesion of the SNpc with lactacystin, and again at weeks 1, 3 and 5, post‐lesioning. To confirm appropriate lactacystin lesioning of the SNpc, any animal which failed to perform ipsilateral rotations in the amphetamine‐induced rotation test (see below) was excluded from further study. No animals were excluded on this basis. Animals received daily injections (i.p.) for 28 days (at the same time each day, 10–11 am) with either saline (Baxter, Newbury, Berkshire, UK, #FKE1324) or doses of nicotinamide (Sigma, Dorset, UK, #N3376) previously shown to have neuroprotective effects in animal models of PD (Anderson *et al*. 2006, 2008) (250 mg/kg or 500 mg/kg in saline), starting at day 7 post lactacystin lesioning. This time point was chosen for initiation of treatment having previously been shown to exhibit significant dopaminergic pathology following lactacystin lesioning (Harrison *et al*. 2015). After final behavioural and MRI measurements, animals were culled, and brains removed for analysis. Treatment and assessment of the control group (Lacta(−)Saline(−)) of animals was performed first followed by experimental groups (Lacta(+)NTA(−), Lacta(+)NTA(+) and Lacta(+)NTA(++)). No randomization was performed.

In addition to the four treatment groups detailed above (Fig. 1), a separate cohort of male Sprague–Dawley rats (250 ± 10 g, *n* = 7) were injected daily for 28 days with the higher dose of nicotinamide used in the study (500 mg/kg, i.p.), to determine the effects of nicotinamide alone on the cellular and molecular readouts reported in the four study treatment groups. After the final i.p. injection on day 28, these animals were culled, and brains removed for analysis. See Figure S1.

### Stereotaxic lesioning of the SNpc with lactacystin

Rats were rendered unilaterally Parkinsonian, by stereotaxic injection of the irreversible proteasome inhibitor, lactacystin (Enzo Life Sciences, Exeter, UK, #BML‐PI104) into the left SNpc, as previously published (Harrison *et al*. 2015). Animal were anaesthetized with isoflurane (Teva, Castleford, UK, #32035519), and positioned in the horizontal skull position into a stereotaxic frame (Kopf Instruments, Tujunga, CA, USA, #900). Isoflurane anaesthesia was chosen for stereotaxic lesioning of the SNpc as previous studies from our group have shown that this regime results in the greatest level of dopaminergic degeneration on SNpc lesioned, compared to other commonly used small animal anaesthetics (Datla *et al*. 2006). Vidine antiseptic scrub (EcoLab, Cheshire, UK. #3030440) was applied to the scalp before a midline incision was made to expose the skull. Bregma was identified (Paxinos and Watson 2009), and above the location of the SNpc, a small burr hole was made. With use of a 10 μL Hamilton syringe (Sigma, #701N), 10 μg of lactacystin (2.5 μg/μL in sterile saline, 4 μL in total) was injected (1 μL/min) at the location of the left SNpc: anterio‐posterior, −5.2 mm, medio‐lateral, +2.5 mm and ventral to dura, −7.6 mm (Paxinos and Watson 2009). The needle was left in place for 3 min prior to being retracted and suturing of the scalp incision. Animals were then left to recover in a heated recovery chamber before being returned to their home cages. In order to minimize animal suffering following stereotaxic surgery a number of measures were taken. Firstly, local anaesthesia, in the form of subcutaneous bupivacaine (Mercury Pharma, London, UK, #02848/0198), was administered at the scalp 3 min prior to making the initial incision. In addition, in order to alleviate post‐operative pain, analgesia, in the form of buprenorphine (Vetergesic; Alstoe Animal Health, York, UK, #117299), was administered intramuscularly prior to recovery. Lastly, post‐surgery fluid replacement (Glucosaline, 5 mL of 0.18% NaCl, 4% Glucose, (Baxter, #FE1253) given i.p. prior to surgery) was given in order to prevent dehydration during recovery. In the days following surgery, sutures were checked for signs of infection, and antibiotic ointment applied (Isaderm; Dechra, Northwich, UK, #570831) daily.

### Behavioural testing

The unilateral lesioning of the nigrostriatal tract in the animals induces an asymmetry in motor function between the left and right‐hand sides of the body, and the extent of the asymmetry is proportional to the extent of the lesion. The vertical cylinder test and amphetamine‐induced rotation test were utilized to quantify motor asymmetry, longitudinally.

#### Vertical cylinder test

The vertical cylinder test (Schallert *et al*. 2000) was used to make longitudinal assessment of forelimb asymmetry in study animals, as previously published (Harrison *et al*. 2015). Briefly, animals were placed into a custom made clear Perspex cylinder (30 cm in height, 20 cm in diameter) and a video camera used to record rearing behaviour for 3 min, or 10 complete rears, whichever came first. In a blinded fashion, offline frame‐by‐frame analysis was used to examine forelimb movements, calculating the % contralateral forelimb use (*N*) as:

$$N=\frac{\left(\text{contralateral forelimb placements}+\frac{1}{2}\;\text{placements of both forelimbs simultaneously})\times 100\right)}{(\text{ipsilateral forelimb placements}+\text{contralateral forelimb placements}+\;\text{placements of both forelimbs simultaneously)}}$$

#### Amphetamine‐induced rotation test

The amphetamine‐induced rotation test (Ungerstedt and Arbuthnott 1970) was used to make longitudinal assessment of rotational asymmetry, as previously published (Harrison *et al*. 2015). Briefly, rats were treated with amphetamine (5 mg/kg (at 5 mg/mL in sterile saline) D‐amphetamine sulphate (Sigma, #A5880), i.p.) and left to acclimatize for 30 min in a clear circular test arena (36 cm in height, 40 cm in diameter, Circling Bowl; Harvard Apparatus, Cambridge, MA, USA, #CMA8309031). Behaviour was then recorded with a video camera for a further 30 min, prior to animals being returned to their home cages. In a blinded fashion, offline quantification of the number of ipsiversive (anticlockwise) and contraversive (clockwise) rotation, in bins of 5 min, was performed, calculating the net ipsiversive rotations (*N*) as:

N=(ipsiversive rotations)−(contraversive rotations)

Further to this, the area under the curve (AUC) produced by plotting number of *N* vs. time, was also calculated as a gross measure of test performance.

### Magnetic resonance imaging

Longitudinal acquisition of *in vivo* magnetic resonance (MR) images of the rat brain was performed in order to quantify and track progression of neuropathology in the lactacystin rat model of PD, as previously published (Harrison *et al*. 2015). Images were acquired on a 4.7 Tesla DirectDrive horizontal small bore MRI scanner (Varian, Palo Alto, CA, USA), with radio frequency transmitted and received using a 72 mm quadrature birdcage head coil (M2M Imaging, Richmond Heights, OH, USA). Animals were anaesthetized in an anaesthetic chamber with isoflurane in O_2_, prior to being transferred into a polytetrafluroethylane MRI compatible head holder, and anaesthesia maintained by delivery of isoflurane in O_2_ via a nose cone. A respiratory balloon (SA Instruments, Stony Brooks, NY, USA, #1030) placed under the animal's thorax, was used to monitor depth of anaesthesia, while a rectal probe and warm air blower (SA Instruments, #1030) were used to monitor and maintain core temperature. A multi‐echo, multi‐slice spin‐echo pulse sequence (MEMS) was used to acquire T2‐weighted images: FOV = 35 mm × 35 mm; matrix = 192 × 192; TR = 5155.2 ms; TE = 10, 20, 30, 40, 50, 60, 70, 80, 90, 100 ms; 4 averages, scan duration 1 h 5 min 59 s. 50 coronal image slices (in plane resolution of 256 × 256 μm), with a thickness of 500 μm, were acquired, covering the entirety of the animal's brain. Post‐acquisition, the ten echo time for each acquired image were summed [‘Z Project’ function in ImageJ (v1.4, National Institutes of Health, Bethesda, MD, USA)] resulting in 50 contiguous T2‐weighted images. Following image acquisition, animals were removed to a heated recovery chamber in order to allow recovery from anaesthesia, before being returned to their home cages.

### MR image analysis

Manual segmentation analysis and also unbiased, automated, tensor‐based morphometry of acquire T2‐weighted images were used to examine changes in neuropathology over time, and to quantify the effects of nicotinamide treatment on lactacystin‐lesioned animals.

#### Manual segmentation analysis

With reference to a rat brain atlas (Paxinos and Watson 2009), delineation of structures (lateral ventricles, corpus striatum, hippocampus and midbrain) was performed manually by a single rater blind to animal treatment (ImageJ), as previously published (Harrison *et al*. 2015). Multiplication of the delineated area (μm^2^) by the slice thickness (500 μm) gave the volume (μm^3^), which was then converted to % change from baseline in order to ascertain changes in volumes over time.

#### Tensor‐based morphometry

We applied tensor‐based morphometry analysis to images acquired from all treatment groups at week 5 post‐lesioning, to search for relative differences in brain volume. For this, structural analysis of MR images brains were oriented to a standard space and intensity non‐uniformity correction applied using the N4ITK algorithm (Tustison *et al*. 2010). Brains were then masked semi‐automatically, by manually masking a single rat brain using ITK‐Snap (Yushkevich *et al*. 2006), prior to other brain images being registered to this masked image using the open‐source NiftyReg package (12 degrees of freedom affine registration, available from: http://sourceforge.net/projects/niftyreg, (Modat *et al*. 2014)). The mask was resampled to each image's space (with nearest neighbour interpolation) using the inverted resulting affine transformation matrices. Intensities between images were standardized within their respective masks, using the method described by Nyúl *et al*. (Nyul *et al*. 2000). All images were then group‐wise registered into a single target ‘atlas’ space (Cleary *et al*. 2011). Group registration attempts to align equivalent anatomical regions between all images, by applying a deformation field. For initial alignment, a single target brain was chosen at random and all other images rigidly registered to it. This was followed by nine iterations of affine registration and 15 iterations of non‐rigid registration, using a symmetric implementation of free‐form deformation (Modat *et al*. 2012). After each iteration, images were resampled into the middle space and their intensity average generated and used as the following iteration's target. Following registration, the Jacobian determinant was calculated at each voxel in the resulting deformation fields. The determinant describes the voxel‐wise expansion or contraction of each image to meet the final average. These were log‐transformed and smoothed (Gaussian kernel, FWHM 0.2 mm). Finally, two‐tailed *t*‐tests were performed at each voxel between all images, using contrasts with the General Linear Model to compare groups’ local volume differences. False Discovery Rate correction was applied (*q* = 0.1) to control for multiple tests (Benjamini and Hochberg 1995). The resulting statistical maps are shown in Fig. 4.

### Tissue collection and preparation

After study completion, animals were culled through inhalation of CO_2_, decapitated and the brain quickly removed out of the skull. The brains were the cut coronally, at the level of the infundibular stem, producing forebrain (containing the striatum) and hindbrain (containing the SNpc) tissue blocks. The frontal block was snap frozen on dry ice and stored, prior to extraction of mRNA and protein, at −80°C. The hind block was drop fixed for 72 h in 4% paraformaldehyde (Sigma, #P6148) in phosphate‐buffered saline (PBS) (pH 7.4), after which it was croyoprotected by submerging in 30% sucrose (Sigma, S9378) in PBS, until the block was observed to have sunk. This tissue was then snap frozen, in pre‐chilled (on dry ice) isopentane (Sigma, #M32631) and stored at −80°C prior to sectioning for immunohistochemical staining.

### Immunohistochemistry

Hind brain tissue was sectioned and immunohistochemically stained as previously published by our group (Harrison *et al*. 2015). Briefly, tissue containing the SNpc was firstly cryosectioned coronally (30 μm thickness) using a cryostat (Bright Instruments, Huntingdon, UK), onto SuperFrost^®^ Plus slides (VWR international, Lutterworth, Leicestershire, UK, #631‐0108), and stored at −80°C prior to analysis. To assess the extent of nigrostriatal dopaminergic cell loss on the lesioned and unlesioned side of the brain, immunohistochemical staining of dopaminergic neurons in the SNpc was utilized. For this, tyrosine hydroxide (TH), an enzyme involved in monoamine synthesis in these neurons, was used as a marker, and cresyl violet used as a counter‐stain in order to label the Nissl body of neurons, as previously published (Harrison *et al*. 2015). Avidin‐Biotin Complex/peroxidase immunohistochemistry was used. Briefly, activity of endogenous peroxidase was firstly blocked by incubating sections in 0.3% H_2_O_2_ (Sigma, #H1009) in methanol (Sigma, #322415) for 45 min. Sections were then put through a descending series of alcohols in order to rehydrate them, before washing in PBS containing 0.1% TritonX‐100 (Sigma, #X100) (PBS‐T). Sections were then blocked for 1 h at 21°C in 20% normal goat serum (VectorLabs, Peterborough, UK, #S‐1000) in PBS‐T, and then incubated in primary TH antibody (1 : 1000 in PBS‐T, Rabbit Polyclonal Anti‐Tyrosine Hydroxylase, #AB152; Millipore Corporation, Bedford, MA, USA, RRID:AB_390204) for 24 h at 21°C. Secondary antibody was applied to sections (1 : 200 in PBS‐T, Biotinylated Goat Anti‐Rabbit Secondary Antibody; VectorLabs, #BA‐1000) for 1 h at 21°C, after washing in PBS‐T. After secondary incubation, tissue was washed again in PBS‐T before being incubated in Avidin‐Biotin Complex (Vectastain Elite ABC Kit; VectorLabs, #PK‐6100) for 1 h at 21°C. Sections were then washed in tris‐buffered saline (TBS) (pH 8.4) before being immunopositive staining was visualized with 3, 3′‐diaminobenzidine (VectorLabs, #SK‐4100). Sections were then finally washed with H_2_O and counter‐stained with cresyl violet (0.1% in dH_2_O) (Sigma, #C5042) prior to dehydration (ascending series of alcohols and xylene) and mounting in Distyrene, dibutyl Phthalate, Xylene (DPX).

### Stereological cell quantification

Stereological (optical fractionator method (West *et al*. 1991)) quantification of the number of TH positive (TH+) and Nissl positive (Nissl+) cells were made in the entire SNpc, with the experimenter blinded to treatment group, as previously published (Harrison *et al*. 2015). For this, a Nikon Eclipse E8 – microscope (Nikon Instruments, Amsterdam, Netherlands), JVC (UK) 3CCD camera, and stereology software system (ImagePro; MediaCybernetics, Rockville, MD, USA) was used. Firstly, an area of interest (AOI) was draw digitally on the section, based on previously published boundaries of the SNpc (Carman *et al*. 1991). Counting frames (140 × 160 μm), were then placed on this AOI using the uniform random sampling method. TH+ and Nissl+ cells within these frames were then counted using ‘acceptance’ (north and west) and ‘forbidden’ (south and east) lines where cells were observed to penetrate these lines, to avoid edge effects. Estimates of total cell numbers (*N*) within the SNpc were calculated using the below equation:

N=n(1/ssf)(1/asf)(1/hsf)

Where the section sampling fraction (*ssf*) is given as 1/6, owing to every 6th section of the SNpc being assessed. The area sampling fraction (*asf*) is given by the total counting frame area relative to the AOI area. And the height sampling fraction (*hsf*) is given by measuring the optical dissector height (average of three random points) height assessed using a Heidenhain microcator (Heidenhain, Traunreut, Germany, #MT60)) relative to the sectioned thickness (30 μm).

### Protein and mRNA extraction and quantification

A tissue homogenizer (Ultra‐Turrax T18; IKA, Oxford, Germany) was used to homogenize 30 mg of frontal brain tissue in QIAzol^®^ Lysis Reagent (Qiagen, Manchester, UK, #79306). The RNeasy^®^ Plus Universal Mini Kit (Qiagen, #73404) was then used for extraction of protein and mRNA, as per the manufacturer's instructions. Extracted protein quantity was measured by Bradford Assay (Sigma, #B6916), determining colour change with a 96‐well plate reader (VersaMax Microplate Reader; Molecular Devices, Palo Alto, CA, USA, #VERSAMAX) at A_595_. Extracted RNA purity and quantity was measured using a NanoDrop ND‐1000 spectrophotometer (Labtech, Heathfield, East Sussex, UK) [mean A_260/280_ ratio = 1.99 (range 1.97–2.01)]. Extracts were stored at −20°C (protein) and −80°C (RNA) until analysis (see below).

#### Protein quantification: western blot analysis

A common acetylation site of H3 (lysine residue 9) (AcH3‐Lys9), was quantified in protein extracts from the frontal brain as a measure of histone acetylation in the brain as previously published (Harrison *et al*. 2015). Briefly, 10 μg of extracted protein sample was denatured (95°C for 15 min) in Laemmli sample buffer (Sigma, #S3401) prior to being loaded onto a hand‐cast 1 mm thick 15% Tris‐Glycine gel and electrophoretically separated (65 mA for 40 min). Proteins were then semi‐dry transferred (20 V for 45 min) onto polyvinylidene difluoride membrane (0.45 μm pore size) soaked in methanol, and equilibrated in TBS containing 0.2% Tween‐20 (TBS‐T). Membrane non‐specific antibody binding was blocked (5% non‐fat milk in TBS‐T, 1 h at 21°C) prior to incubation of membranes with primary antibodies for both AcH3‐Lys9 (rabbit anti‐AcH3‐Lys9, 1 : 10 000; Sigma, #H9286, RRID:AB_477076) and β‐actin as a loading control (mouse anti‐β‐actin, 1 : 20 000; Abcam, Cambridge, UK, #Ab6276, RRID:AB_2223210) for 1 h a 21°C. After washing, membranes were incubated with horseradish peroxidase‐conjugated secondary antibodies (either Goat anti‐Rabbit, 1 : 10 000, and Horse anti‐Mouse, 1 : 10 000, for AcH3‐Lys9 or β‐actin, respectively, both VectorLabs, #PI‐1000 and #PI‐2000) for 1 h at 21°C, prior to positive signal being visualized and developed onto photographic film using chemiluminescence (Clarity Western ECL Substrate; Bio‐Rad, Watford, Hertfordshire, UK, #1705060). Protein bands were quantified densitometrically (ImageJ, v1.4).

#### mRNA quantification: quantitative real‐time polymerase chain reaction

In addition to extraction of protein for quantification of histone acetylation, mRNA was also extracted from excised brain samples for quantification of the expression levels of *SNCA*, brain‐derived neurotrophic factor (*BDNF*), glial‐derived neurotrophic factor (*GDNF*), *HSPA1A*,* GSN*,* BCL2* and *BAD,* as previously published by our group (Harrison *et al*. 2015), as changes in the expression levels of the proteins corresponding to these genes have been previously to be associated with HDACI treatment (Monti *et al*. 2009). Briefly, cDNA was synthesized from extracted mRNA, by reverse transcription of 500 ng total RNA using the QuantiTect^®^ reverse transcription kit (Qiagen, #205311) with integrated removal of genomic DNA contamination. This was then quantified using real‐time reverse transcriptase quantitative polymerase chain reaction (RT‐qPCR) on an Mx3000P™real‐time PCR system with MxPro software (v4.10; Stratagene, La Jolla, CA, USA). Duplex reactions were set up in triplicate, containing 10 μL of 2× Brilliant^®^ II QPCR master mix (Agilent Technologies UK Ltd, Stockport, Cheshire, UK, #600806), 1 μL of 10× PrimeTime™qPCR assay for the novel reference gene X‐prolyl aminopeptidase (aminopeptidase P) 1 (XPNPEP1) (Durrenberger *et al*. 2012), 1 μL of 10× PrimeTime™qPCR assay for the gene of interest, 7 μL RNase free H_2_0 and 1 μL of sample cDNA. For full probe and primer sequences of PrimeTime™ qPCR assays (Integrated DNA Technology, Coralville, IA, USA), see table 1. Reactions were activated at 95°C for 10 min, followed by running of 60 cycles of a three step thermocycler program (95°C for 30 s, 55°C for 30 s and 72°C for 30 s), acquiring fluorescence data at the annealing step. Normalized relative expression was computed from C_T_ values using the 2^−ΔΔCT^ method (Livak and Schmittgen 2001), normalizing to the appropriate control group.

**Table 1:** Probe and primer sequences of PrimeTime™ qPCR assays used

Gene	Protein	Sequence	
XPNPEP1	XPNPEP1	Probe	5′‐/5HEX/CCATCATTC/ZEN/ACTACGCGCCGATCC/3IABkFQ/‐3′
		Primer 1	5′‐GTTCCATCCTTGTACTGAGCA‐3′
		Primer 2	5′‐TTCCCAACGATTTCCAGCA‐3′
SNCA	α‐Synuclein	Probe	5′/56‐FAM/CTTCTCAGC/ZEN/CACTGTTGTCACTCCA/3IABkFQ/‐3′
		Primer 1	5′‐CCCTCCAACATTTGTCACTTG‐3′
		Primer 2	5′‐GCGTCCTCTATAGGTTCCA‐3′
BDNF	BDNF	Probe	5′‐/56‐FAM/CAGCAAAGC/ZEN/CACAATGTTCCACCA/3IABkFQ/‐3′
		Primer 1	5′‐GCAACCGAAGTCTGAAATAACCA‐3′
		Primer 2	5′‐GACACATTACCTTCCAGCATCT‐3′
GDNF	GDNF	Probe	5′‐/56‐FAM/CGCTGACCA/ZEN/GTGACTCCAATATGCC/3IABkFQ/‐3′
		Primer 1	5′‐CAGTCTTTTGATGGTGGCTTG‐3′
		Primer 2	5′‐GCCGAAGACCACTCCCT‐3′
HSPA1A	HSP70	Probe	5′‐/56‐FAM/CCGTGTTGT/ZEN/GGACAGTTGGTTGTG/3IABkFQ/‐3′
		Primer 1	5′‐TGAGTGGAATGGACAGGAAAG‐3′
		Primer 2	5′‐CATAATCAGAACTGTGCGAGTCT‐3′
GSN	Gelsolin	Probe	5′‐/56‐FAM/CGCCAGGAA/ZEN/CCTCTTCGATCACAA/3IABkFQ/‐3′
		Primer 1	5′‐CATCAGTAGCCAGGTCTTCC‐3′
		Primer 2	5′‐GGCTTAAGGACAAGAAGATGGA‐3′
BCL2	BCL2	Probe	5′‐/56‐FAM/CAGGATAAC/ZEN/GGAGGCTGGGATGC/3IABkFQ/‐3′
		Primer 1	5′‐CCAGGAGAAATCAAACAGAGGT‐3′
		Primer 2	5′‐GATGACTGAGTACCTGAACCG‐3′
BAD	BAD	Probe	5′‐/56‐FAM/CCATAGTCC/ZEN/CAGCCCTCCATG/3IABkFQ/‐3′
		Primer 1	5′‐CATCCCTTCATCTTCCTCAGTC‐3′
		Primer 2	5′‐GACAGGCAGCCAATAACAGT‐3′

All PrimeTime™ qPCR assays were obtained from Integrated DNA Technology (Coralville, IA, USA) and contained 2.5 nM of probe, 5 nM of primer 1 and 5 nM of primer 2. A, adenine; C, cytosine; G, guanine; T, thymine; HEX™, Hexachlorofluorescein; IABkFQ, Iowa Black^®^ FQ.

### Statistical analysis

All data are presented as either mean ± standard error of mean between animals in line graphs, or as box‐plots; with the middle line representing the median, the box representing the 25th to 75th percentiles and whiskers presenting the minimum and maximum values, with a + representing the mean in each box. No calculation was performed to predetermine the sample size, nor was any test for outliers. Prior to between‐group statistical analysis, data normality was confirmed using the Kolmogorov–Smirnov test. Vertical cylinder test, amphetamine‐induced rotation test and MRI manual segmentation datasets were analysed using two‐way (repeated measures) analysis of variance (anova) tests with Bonferroni post‐tests. In addition, a paired t‐test was used to compare the baseline and week 1 forelimb use of lactacystin‐lesioned animals in vertical cylinder test data. Differences between stereological cell counts in the ipsilateral and contralateral hemispheres of animal brains were tested using paired *t*‐tests, with a one‐way anova with Bonferroni post‐tests used to compare percentages of cell loss from counts. A two‐way anova with Bonferroni post‐tests was used to compare qRT‐PCR data, and lastly a one‐way anova with Bonferroni post‐tests was used to compare western blot data. All statistical analyses was performed using GraphPad Prism (v5.0 for Windows; GraphPad Software Inc., San Diego, CA, USA).

## Results

### Nicotinamide exacerbates behavioural motor deficits caused by lactacystin

#### Vertical cylinder test

**Figure 2:**
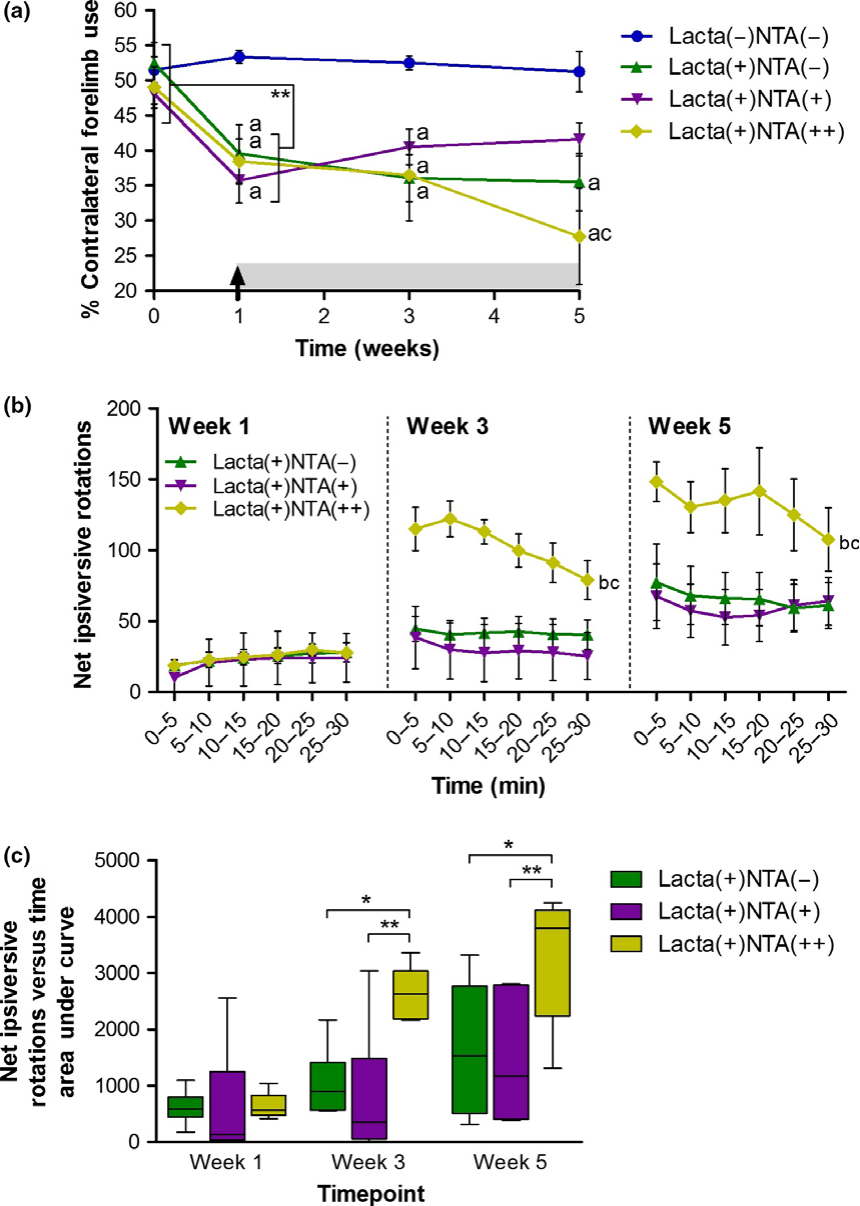
High doses of nicotinamide exacerbate behavioural motor deficits caused by lactacystin lesioning of the Substantia Nigra pars compacta. (a) Vertical cylinder test outcomes show that once animals begin treatment with nicotinamide at week 1 (designated by arrow and grey shading) the lactacystin‐induced reduction in percentage contralateral forelimb use is slightly alleviated upon treatment with a low dose (250 mg/kg) of nicotinamide, however, exacerbated upon treatment with a high dose (500 mg/kg) of nicotinamide, compared to saline‐treated control animals. (b and c) Amphetamine‐induced rotation test outcomes show that animals treated with the low dose of nicotinamide (250 mg/kg) perform slightly fewer rotations over time, whereas animals treated with the high dose of nicotinamide (500 mg/kg) exhibit far greater rotational behaviour as a result of drug treatment. Statistical significance denoted with either asterisks: (*p < 0.05, **p < 0.01) or letters where *p* < 0.05 for each comparison: ^a^Significantly different from group Lacta(−)NTA(−); ^b^Significantly different from group Lacta(+)NTA(−); ^c^Significantly different from group Lacta(+)NTA(+). Data are presented as mean ± SEM, or as box‐plots, with the middle line representing the median, the box representing the 25th to 75th percentiles and whiskers presenting the minimum and maximum values, with a + representing the mean in each box. *n* = 6–7 animals per group.

At baseline there was equal use of both the left and right forelimb in all animals (Fig. 2a). However, 1 week after surgery there was a significant asymmetry in forelimb paw usage as evidenced by a reduction in the contralateral forelimb use of lactacystin‐lesioned animals compared with non‐lesioned animals (paired *t*‐test, *p* < 0.01). Contralateral forelimb use of the lactacystin‐lesioned, saline‐treated animals continued to decline with time over the 5 week study period. However, in lactacystin‐lesioned animals subsequently treated with the low dose of nicotinamide starting at day 7 post‐lesioning, a slight alleviation of this motor behavioural deficit was observed at weeks 3 and 5: contralateral forelimb use in this animal treatment group after 28 days of nicotinamide (250 mg/kg) treatment recovering by 5.87 ± 2.39% (*p* > 0.05 compared to non‐lesioned animals at week 5). This markedly contrasted to the animals treated with the high dose of nicotinamide, where the contralateral forelimb use continued to decline with time but at a faster rate than that observed in the saline‐treated lactacystin‐lesioned animals; resulting in a further 8.92 ± 8.08% reduction in contralateral forelimb use after 28 days of nicotinamide (500 mg/kg) treatment (*p* < 0.001 compared to non‐lesioned animals, and *p* < 0.05 compared to low‐dose nicotinamide‐treated animals, at week 5).

#### Amphetamine‐induced rotation test

Ipsilateral rotations following an amphetamine challenge were observed at 1 week in all lactacystin‐lesioned animals (Fig. 2b). Saline‐treated lactacystin‐lesioned animals performed increasing numbers of rotations over the further two time points examined (Fig. 2c, AUC produced by plotting number of rotations vs. time; week 1, 612.08 ± 121.59; week 3, 1039.33 ± 247.75; week 5, 1641.00 ± 477.50). Treatment of animals with a low dose of nicotinamide (250 mg/kg) did not markedly attenuate the number of amphetamine‐induced rotations observed in later time points of the study. However, animals subsequently treated with a high dose of nicotinamide exhibited a marked exacerbation in the number of amphetamine‐induced rotations, observed at both weeks 3 and 5 (AUC produced by plotting number of rotations vs. time, *p* < 0.05 and *p* < 0.01 compared to saline‐treated and low‐dose nicotinamide‐treated lactacystin‐lesioned animals, respectively, at both 3 and 5 weeks post‐lesion).

### MRI reveals dose‐dependent exacerbation of lactacystin‐induced volumetric changes by nicotinamide

#### Manual segmentation analysis

**Figure 3:**
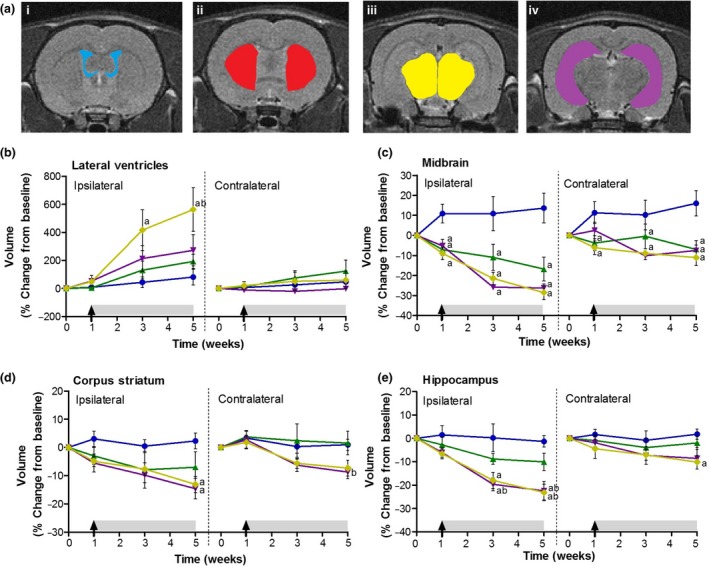
Manual segmentation analysis of MR images reveals exacerbation of lactacystin‐induced volumetric changes upon treatment with nicotinamide. (a) Representative examples of the manual segmentation of (i) lateral ventricles, (ii) corpus striatum, (iii) midbrain and (iv) hippocampus. Administration of nicotinamide (designated by arrow and grey shading) exacerbates volumetric changes observed in the (b) lateral ventricles, the (c) midbrain, the (d) corpus striatum and the (e) hippocampus, as a result of lactacystin lesioning, as ascertained through manual segmentation analysis of rat brain MR images. Statistical significance denoted with letters where *p* < 0.05 for each comparison: ^a,^ significantly different from group Lacta(−)NTA(−); ^b^Significantly different from group Lacta(+)NTA(−) Data are presented as mean ± SEM. *n* = 6–7 animals per group.

In surgically naïve animals, the volume of the lateral ventricles increases comparably in both hemispheres over the 5 week study (Fig. 3b). However, lactacystin‐lesioned animals subsequently treated with saline, exhibited a greater increase in ventricular enlargement over the 5 weeks of study, a change which was more pronounced in the lesioned hemisphere. Nicotinamide treatment of animals resulted in a dose‐dependent exacerbation of ipsilateral ventricular enlargement over the study period, resulting in animals treated with the high dose (500 mg/kg) of nicotinamide exhibiting significantly larger ipsilateral ventricles than both surgically naïve, and lactacystin‐lesioned saline‐treated animals by week 5 (Fig. 3b, *p* < 0.001 and *p* < 0.05 respectively). Unsurprisingly, given the location of the SNpc in the midbrain, and the injection of lactacystin into this brain region to produce this animal model of PD, the midbrain was the area observed to be most markedly affected in MR images. One week after lesioning surgery, prior to commencement of saline/nicotinamide treatment, the ipsilateral midbrain volume of all lactacystin‐lesioned animal groups was significantly lower than the volume of the surgically naïve group (Fig. 3c, lactacystin‐lesioned saline, 250 mg/kg and 500 mg/kg nicotinamide‐treated animals, 7.07 ± 3.05%, 5.20 ± 3.14% and 8.94 ± 3.11% decrease from baseline, respectively, vs. 10.88 ± 4.73% increase from baseline in the surgically naïve group, *p* < 0.05 in all comparisons). The midbrain volume of the group of animals lesioned with lactacystin and subsequently treated with saline, continued to decline over the further two imaging time points (week 3, 10.90 ± 6.54% decrease from baseline; week 5, 16.77 ± 5.94% decrease from baseline, *p* < 0.01 and *p* < 0.001 compared to surgically naïve animals at week 3 and 5 respectively). The midbrain volume of both lactacystin‐lesioned nicotinamide‐treated animal groups (250 mg/kg and 500 mg/kg) also continued to decline over the subsequent two imaging time points. However, this decline in midbrain volume was far greater in both of these groups compared to saline‐treated lactacystin‐lesioned animals (Fig. 3c, week 3, saline‐treated animals, 10.90 ± 6.54% decrease from baseline, compared to low‐ and high‐dose nicotinamide‐treated animals, 25.81 ± 0.99% and 21.40 ± 3.04% decrease from baseline respectively; week 5, saline‐treated animals, 16.77 ± 5.94% decrease from baseline, compared to low‐ and high‐dose nicotinamide‐treated animals, 26.23 ± 1.99% and 28.56 ± 3.42% decrease from baseline respectively). Identical albeit more subtle changes were similarly observed in the contralateral hemisphere of the midbrain (Fig. 3c). Similarly, identical albeit more subtle changes in volume decreases over time to those observed in the midbrain were seen in both the corpus striatum and hippocampus (Fig. 3d and e).

#### Tensor‐based morphometry

**Figure 4:**
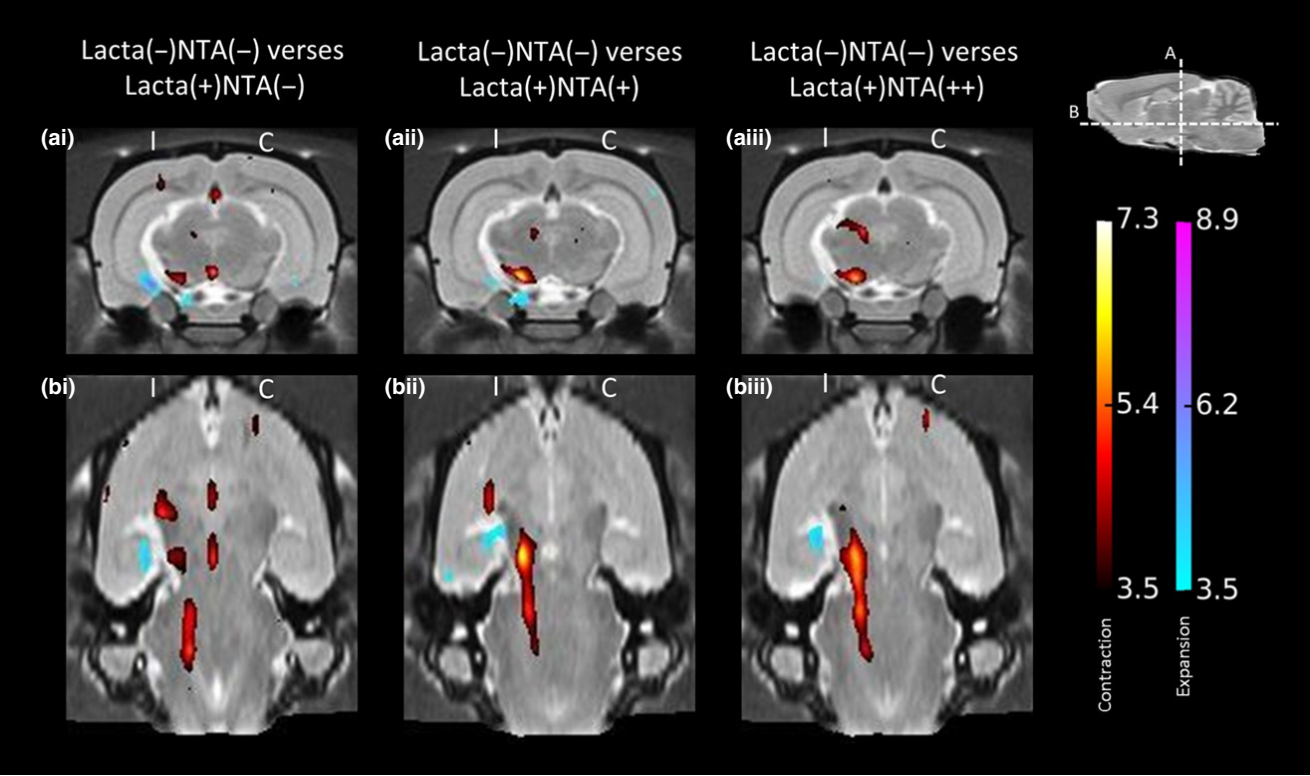
Tensor‐based morphometry validates findings from manual segmentation analyses of rat brain MR images. Regions of significant volume difference for each treatment group compared with Lacta(−)NTA(−) at week 5 are shown, overlaid onto representative slices of the group average image. Positive differences (blue) indicate local volume increases in group compared with Lacta(−)NTA(−) and negative differences (red) indicate local volume decreases in group compared with Lacta(−)NTA(−). Two orientations are shown at the (a) coronal and (b) horizontal levels of the Substantia Nigra pars compacta. Results shown are significant after correction for multiple comparisons across voxels using the False Discovery Rate with *q* = 0.1. *n* = 6–7 animals per group. C, contralateral; I, ipsilateral.

Several distinct anatomical patterns were observed across treatment groups (all data shown are corrected for multiple comparison over voxels using the False Discovery Rate with *q* < 0.1). When comparing the surgically naïve group of animals to saline‐treated lactacystin‐lesioned animals, a number of clusters of significant voxels were observed (Fig. 4ai and bi). Notably, as would be expected, shrinkage was observed in the Substantia Nigra, both in the pars compacta and pars reticulata, accompanied by a cluster of expanding voxels in the neighbouring ventricle. Primarily this reflected an increase in cerebrospinal fluid signal accompanying deformation of the ventral midbrain. A small cluster of contracting voxels was also observed in the superior colliculus, and on the midline, contraction was also observed in pineal recess and more ventrally, in the interpeduncular nucleus. From the horizontal level of the SNpc (Fig. 4bi), significant contraction can also be observed rostral and caudal to the site of lactacystin lesioning: in the optic tract, and in the peduncular pontine and pontine reticular nuclei. These data are consistent with prior observations in this model (Vernon *et al*. 2011; Harrison *et al*. 2015).

When comparing the surgically naïve group of animals to low‐dose (250 mg/kg) nicotinamide‐treated lactacystin‐lesioned animals, a similar, yet enhanced pattern of contraction was observed compared to the drug naïve group comparison (Fig. 4aii and bii). The contractions observed in the midline when comparing surgically naïve to lactacystin‐lesioned saline‐treated animals were absent. Yet a greater number of significantly contracted voxels were observed in the superior colliculus, and also the SN, which again was accompanied by enlargement of the neighbouring ventricle. Similar to the non‐drug‐treated comparison, this contraction in the SN was also observed to be extended caudally into the pontine nuclei, yet to a greater degree.

In animals lesioned with lactacystin and subsequently treated with the high dose (500 mg/kg) of nicotinamide, an even greater extent of contraction was observed compared to the surgically naïve treatment group (Fig. 4aiii and biii). Firstly, a greater number of significantly contracted voxels were observed in the superior colliculus, extending laterally into the pretectal nucleus and the optic tract. Similarly, a larger cluster of significantly contracted voxels was observed in the SN, which again, from the horizontal orientation was observed to extent into the pontine nuclei.

### Nicotinamide treatment causes dose‐dependent exacerbation of dopaminergic neurodegeneration in the SNpc of lactacystin‐lesioned animals

**Figure 5:**
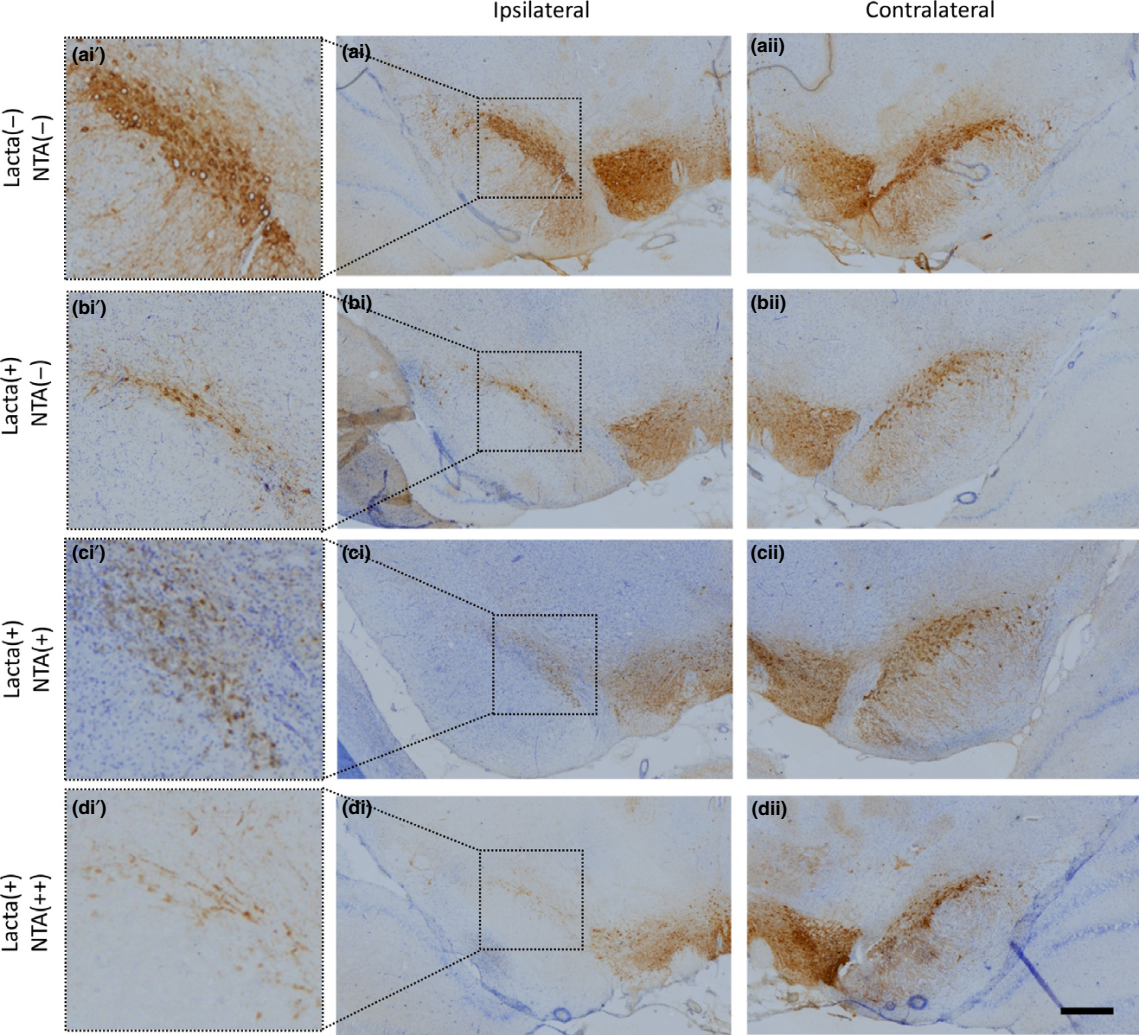
Neurodegeneration in the Substantia Nigra pars compacta (SNpc) of lactacystin‐lesioned animals. Representative examples of the TH and Nissl stained ipsilateral (ai–di) and contralateral (aii–dii) SNpc of rats in each of the four treatment groups. Insets (ai’–di’), allow closer morphological examination of the degenerating SNpc in each treatment group. Scale bar equal to 500 μm.

**Figure 6:**
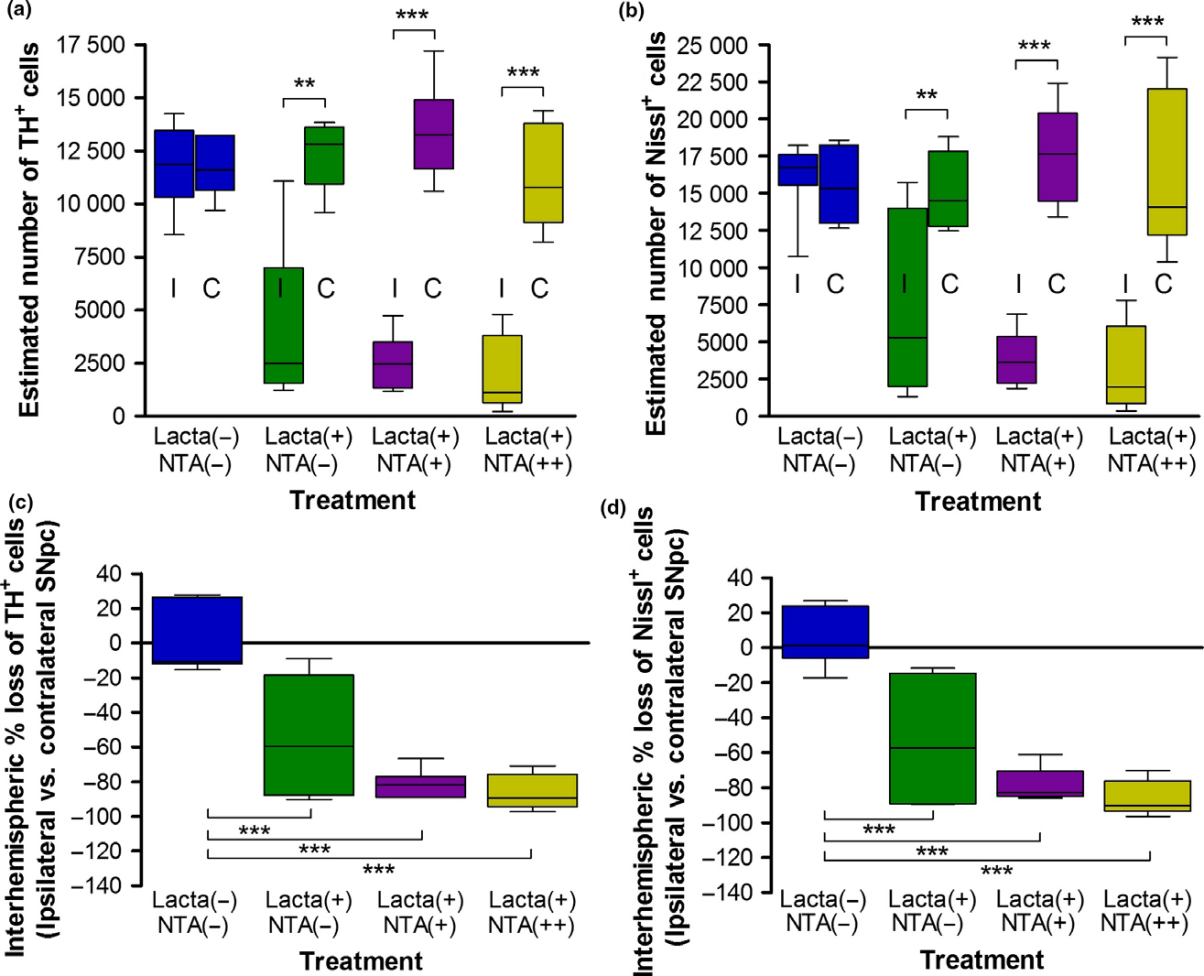
Nicotinamide treatment causes exacerbation of dopaminergic neurons in the Substantia Nigra pars compacta (SNpc) in lactacystin‐lesioned animals. Stereologically estimated (a) TH+ and (b) Nissl+ neuron numbers in the SNpc of rats suggest that nicotinamide exacerbates dopaminergic neurodegeneration in the lactacystin‐lesioned rat brain. This is exemplified by the percentage interhemispheric loss of TH+ (c) and Nissl+ (d) neurons calculated between hemispheres of the SNpc. Statistical significance indicated with asterisks: **p < 0.01, ***p < 0.001. Data are presented as box‐plots, with the middle line representing the median, the box representing the 25th to 75th percentiles and whiskers presenting the minimum and maximum values, with a + representing the mean in each box. *n* = 6–7 animals per group. C, contralateral; I, ipsilateral.

Post‐study, animals were culled and hind brain tissue collected for immunohistochemical staining and stereological quantification of dopaminergic neurons (TH+ and Nissl+) in the SNpc (Figs 5 and 6). No neuronal loss between hemispheres was observed in non‐lesioned animals (Fig. 6a, non‐lesioned animals, left hemisphere, 11724 ± 729, right hemisphere, 11652 ± 493, *p* > 0.05). Animals lesioned with lactacystin and subsequently treated with saline, however, exhibited a marked interhemispheric loss of TH+ neurons, 53.81 ± 13.26%. This loss was dose‐dependently exacerbated with nicotinamide treatment (Fig. 6c and d, 81.41 ± 3.35% and 85.87 ± 4.63% interhemsipheric loss in low (250 mg/kg) and high (500 mg/kg) dose nicotinamide‐treated animals respectively). All changes observed in SNpc TH+ cell number were reproduced in the numbers of Nissl+ cells, indicative of TH+ neuronal cell death rather than loss of the TH enzyme expression in dying neurons. Additionally, upon closer examination of the degenerating SNpc in nicotinamide‐treated cohorts, TH+ neurons in animals treated with the low dose of nicotinamide appear to retain their defined TH+ neuronal cell body structure, yet in rats treated with the high dose of nicotinamide, this structure appears lost: more sparse, diffuse and dendritic TH staining is observed (Fig. 5di’), indicative of the decline in dopaminergic neuronal health in this treatment group.

### Nicotinamide reverses lactacystin‐induced reduction in histone acetylation resulting in histone hyperacetylation in the brain

**Figure 7:**
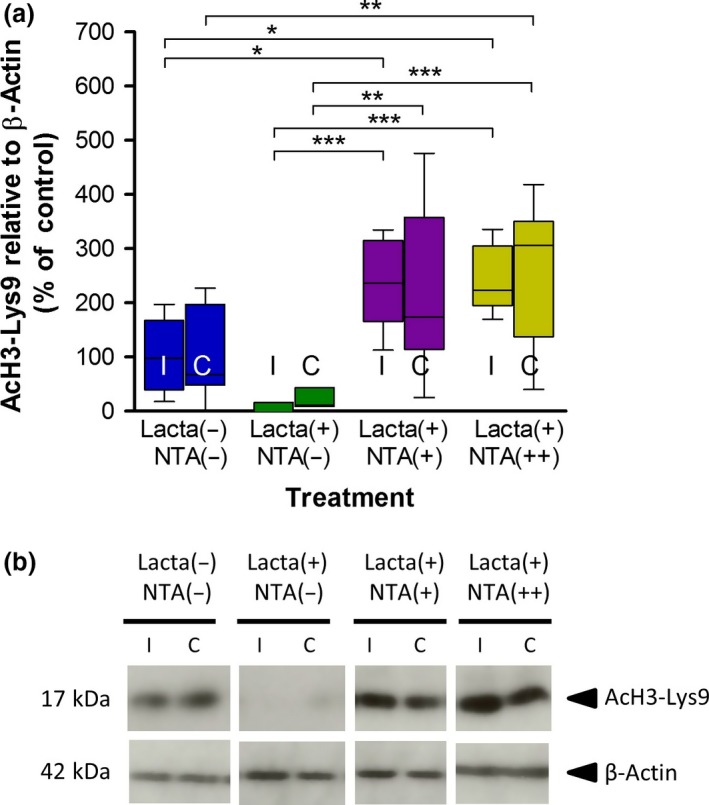
Nicotinamide attenuates lactacystin‐induced reduction in brain histone acetylation. Administration of systemic nicotinamide reverses the reduction in histone H3‐lysine 9 acetylation caused by lactacystin. (a) Densitometry analysis of the AcH3‐Lys9 band relative to the β‐actin band used as a loading control. (b) Representative blot of data presented in (a). Statistical significance indicated with asterisks: *p < 0.05; **p < 0.01, ***p < 0.001. Data are presented as box‐plots, with the middle line representing the median, the box representing the 25th to 75th percentiles and whiskers presenting the minimum and maximum values, with a + representing the mean in each box. *n* = 6–7 animals per group. C, contralateral; I, ipsilateral.

Upon removal of brain tissue at the end of the study, the frontal brain was snap frozen for subsequent quantification of histone acetylation through quantification of AcH3‐Lys9 using Western blot analysis (Fig. 7). A marked reduction in AcH3‐Lys9 was observed in both hemispheres of lactacystin‐lesioned animals treated with saline compared to non‐lesioned animals treated with saline, however, this change was non‐significant. This lactacystin‐induced effect of histone hypoacetylation, however, was reversed upon treatment with nicotinamide, not only back to the levels observed in non‐lesioned animals, but more than two‐fold greater, resulting in significant histone hyperacetylation in nicotinamide‐treated animals’ brains (*p* < 0.01 in all comparisons).

### Nicotinamide dose‐dependently up‐regulates expression of neuroprotective proteins

**Figure 8:**
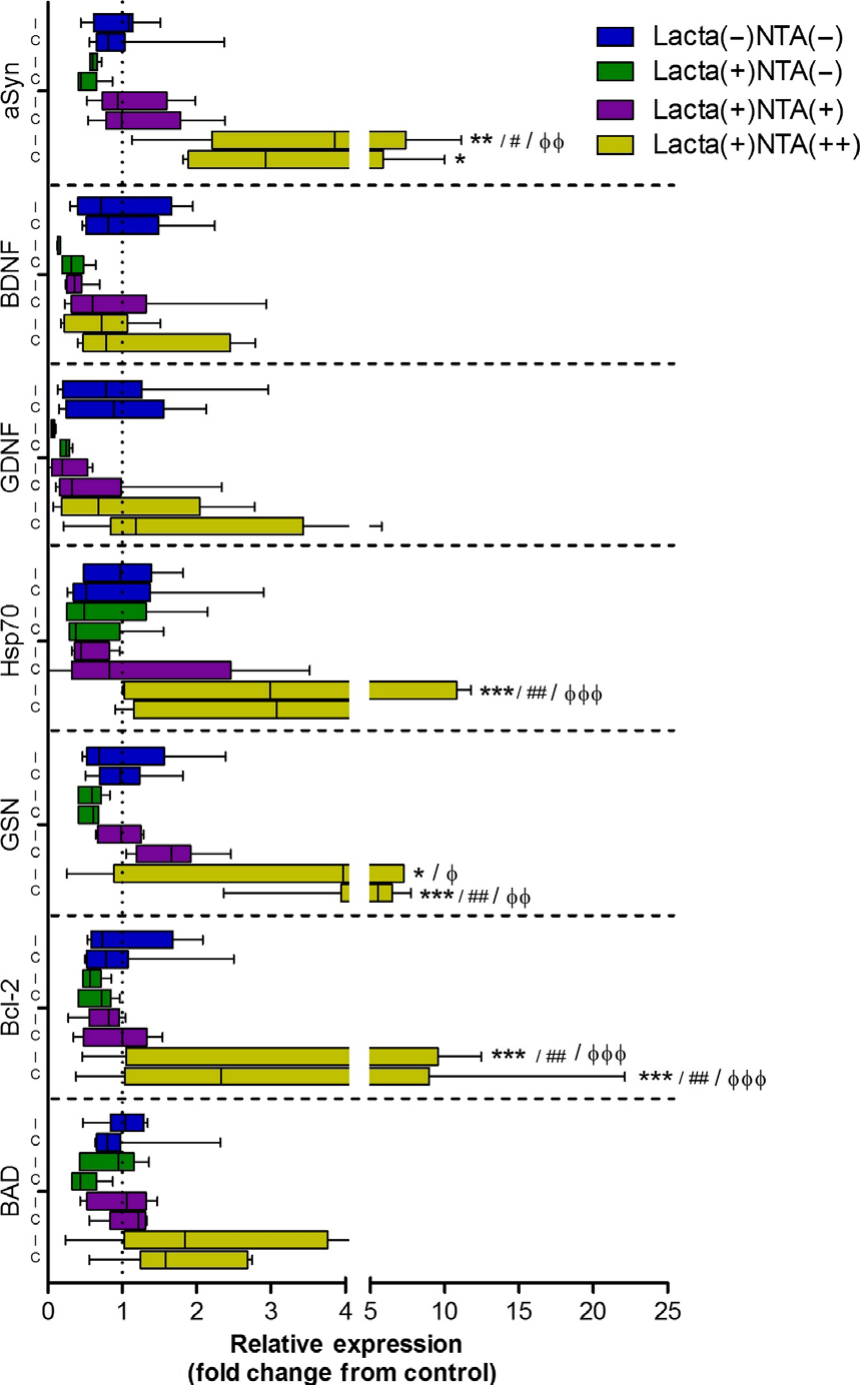
Nicotinamide dose‐dependently up‐regulates expression of neuroprotective and neurotrophic growth factor mRNA in the brain. Lactacystin‐lesioning induced reduction in mRNA expression of αSyn, BDNF, glial‐derived neurotrophic factor (GDNF), Hsp70, GSN, Bcl‐2 and BAD. These reductions were dose‐dependently alleviated upon treatment with nicotinamide. Statistical significance indicated with either asterisk, hash or phi: *p < 0.05, **p < 0.01, ***p < 0.001 compared with the same hemisphere of Lacta(−)NTA(−) group; ^#^ *p* < 0.05, ^##^ *p* < 0.01, compared with the same hemisphere of Lacta(+)NTA(−) group; ^φ^ *p* < 0.05, ^φφ^ *p* < 0.01, ^φφφ^ *p* < 0.001 compared with the same hemisphere of Lacta(+)NTA(+) group. Data are presented as box‐plots, with the middle line representing the median, the box representing the 25th to 75th percentiles and whiskers presenting the minimum and maximum values, with a + representing the mean in each box. *n* = 6–7 animals per group. C, contralateral; I, ipsilateral.

In lactacystin‐lesioned animals treated with saline, there was a marked decrease in the expression of a number of neurotrophic and neuroprotective factors in the brain, such as αSyn, brain‐derived neurotrophic factor, GDNF, gelsolin and Bcl‐2 (Fig. 8). These decreases, however, were not significant. Upon treatment of lactacystin‐lesioned animals with nicotinamide, however, a dose‐dependent attenuation of a number of these reductions were observed: high dose (500 mg/kg) nicotinamide‐treated animals displaying significantly greater expression levels of αSyn, Hsp70, gelsolin and Bcl‐2. These increased expression levels, however, were far greater in animals treated with the higher dose of nicotinamide, disproportionate to the dose of nicotinamide animals were treated with.

## Discussion

Nicotinamide dose‐dependently exacerbated degeneration of dopaminergic nigral neurons in the lactacystin rodent model of PD. This is evident from exacerbation of deficits in motor behaviour, brain volume changes assessed using longitudinal MRI, and *ex vivo* assessment of dopaminergic neuron cell numbers in the SNpc. Molecular analyses indicated that nicotinamide not only reversed lactacystin‐induced histone hypoacetylation in the brain but also induced histone hyperacetylation in nicotinamide‐treated animal brains. This translated to dose‐dependent up‐regulations of numerous neuroprotective protein genes; however, these did not result in neuroprotection in this model of neurodegenerative disease, rather exacerbated neurodegeneration. Careful interpretation of these results and comparison to previously published similar datasets will help to understand how and why, nicotinamide, a HDACI which has been previously shown to act neuroprotectively in models of PD, exacerbates neurodegeneration in the lactacystin rat model of the disease.

Nicotinamide has received increased interest in neurodegenerative disease research in recent years because of it being previously shown to reduce infarct size and resultant neurological deficits in models of stroke (Ayoub *et al*. 1999; Mokudai *et al*. 2000; Liu *et al*. 2009), improve cognition in Alzheimer's disease transgenic mice (Green *et al*. 2008), and up‐regulate neurotrophic factors in models of Huntington's disease leading to improved motor scores (Hathorn *et al*. 2011). Additionally, in contrast to the results observed here, nicotinamide has also previously been shown to act neuroprotectively in animal models of PD (Anderson *et al*. 2006, 2008). In previous published mouse studies using 1‐methyl‐4‐phenyl‐1,2,3,6‐tetrahydropyridine (MPTP), a mitochondrial complex 1 inhibitor used to cause neurodegeneration selectively within the SNpc by way of energy starvation and free radical production, nicotinamide demonstrated a neuroprotective phenotype. Peripheral administration of nicotinamide before MPTP injection, resulted in dose‐dependent neuroprotection in the ‘acute’ MPTP (four injections in 1 day at 2 h intervals) but not in the ‘sub‐acute’ model (two injections per day at 4 h intervals for 5 days) of PD. In this ‘sub‐acute’ model, subtle neuroprotection was observed, but nowhere near that shown in the ‘acute’ dosing strategy (Anderson *et al*. 2006, 2008). These findings are in direct contrast to those presented here. The key difference between these previous studies and that presented here is the toxin used to induce dopaminergic degeneration within the SNpc. Nicotinamide is a precursor of NAD+ and is therefore, in addition to its effects as a HDACI, thought to be involved with brain energy metabolism and preservation of mitochondrial functionality: an increasingly attractive prospect for neuroprotection in PD (Beal 2003). MPTP works through inhibition of mitochondrial complex 1 to cause mitochondrial dysfunction and energy starvation in dopaminergic neurons. It may well be likely then that nicotinamide, in the MPTP model, would directly counteract the effects of this mitochondrial toxin because of its effects on bioenergetics, hence translating to a neuroprotective phenotype observed in the studies by Anderson *et al*. (2006, 2008). Energy starvation and free radical production, however, are by no means the only mechanisms through which dopaminergic neurodegeneration is thought to take place in PD, rather a complex interaction of several pathways, with αSyn protein deposition at the centre. To provide a more complete understanding of the potential of nicotinamide as a neuroprotective agent in PD then, its effects on other mechanistically distinct animal models of the disorder must be investigated.

More akin to the neuropathological picture of degeneration in clinical PD, SNpc toxicity in the rat brain was induced in this study through protein deposition, thought to be the driving force behind epigenetic dysregulation of histone acetylation in PD. This was achieved through SNpc injection of the irreversible proteasome system inhibitor, lactacystin, shown previously to induce αSyn protein deposition in residing dopaminergic neurons (McNaught *et al*. 2002; Fornai *et al*. 2003; Miwa *et al*. 2005; Niu *et al*. 2009; Vernon *et al*. 2010, 2011; Xie *et al*. 2010; Lorenc‐Koci *et al*. 2011; Du *et al*. 2014; Bentea *et al*. 2015; Pienaar *et al*. 2015). Rather than recapitulating the neuroprotective phenotype observed in the MPTP mouse model, nicotinamide appeared to act in the opposing direction in this animal model; exacerbating the effects of lactacystin‐induced nigrostriatal degeneration in the rat brain. When given in isolation to healthy control rats, the high dose of nicotinamide used in this study (500 mg/kg) for the same time period (28 days) had no marked effect on nigrostriatal integrity (see Figure S1). It induced subtle increases in histone acetylation, and equally subtle changes in gene expression (see Figure S1). When given after lactacystin lesioning of the SNpc, however, we observed that nicotinamide‐induced exacerbation of SNpc degeneration, histone hyperacetylation and dramatic increases in gene expression. It has been demonstrated previously *in vitro*, that other HDACIs possess the ability to suppress the activity of the ubiquitin–proteasome system and expression of its subunits (Mitsiades *et al*. 2004). It may well be the case that in this study the toxic effects of nicotinamide after lactacystin lesioning are because of a synergistic inhibitory effect on proteasome activity, providing a potential explanation of the detrimental effects of the drug observed here. That being said, drug/toxin interactions can largely be avoided with the use of a delayed start treatment strategy, unlike the study design used by Anderson *et al*., in their characterization of the effects of nicotinamide in the MPTP model, in which drug administration was commenced prior to toxin administration. In this study, however, drug treatment was commenced 7 days after lactacystin lesioning, meaning that the therapeutic was applied to an already degenerating nigrostriatal system, mimicking more closely the clinical scenario. It may well also be possible then, that by this time, any neuroprotective potential of nicotinamide is outweighed by the extent of further proteasomal inhibition, as a result of lactacystin‐induced protein deposition within dopaminergic neurons; nicotinamide then acting to synergistically exacerbate toxicity via further activity suppression and reducing the expression of ubiquitin proteasome system subunits (Mitsiades *et al*. 2004).

Acetylation of histone lysine residues cause neutralization of their charge, and reduced electrostatic interaction between the phosphate group on DNA and the histone tail itself. Relaxation of chromatin structure then results, as a function of the disrupted histone and DNA inter‐ and intra‐nucleosomal interactions, allowing transcription factors to access DNA. In our previous work, we showed that alleviation of histone hypoacetylation in the lactacystin rat model, to near healthy levels with the use of the class I and IIa HDACI valproate, resulted in neuroprotection (Harrison *et al*. 2015). Here, however, nicotinamide treatment, rather than lessening the degree of histone hypoacetylation, completely reversed the acetylation status in the brain, resulting in extensive histone hyperacetylation in nicotinamide‐treated animals. Such a scenario has previously been observed to be neurotoxic in dopaminergic neurons (Song *et al*. 2010). Consistent with histone acetylation though, large dose‐dependent up‐regulations of neuroprotective and neurotrophic genes were observed in the brains of nicotinamide‐treated rats. In contrast to our previous findings with valproate, however, in which drug‐induced up‐regulation of neurotrophic factors (namely BDNF and GDNF) translated to neuroprotection in the lactacystin rat model of PD (Harrison *et al*. 2015, 2016), exacerbation of pathology rather than protection is observed in this study. It is important to note, however, that the level of histone acetylation observed here upon treatment with nicotinamide was far greater than that observed previously with valproate treatment of the same animal model. It is therefore likely that extensive histone hyperacetylation‐induced chromatin relaxation, like that observed here with nicotinamide, would not only induce up‐regulations of neuroprotective and neurotrophic genes, such as those studied here, but also an array of other genes, many of which possibly pro‐death or pro‐inflammatory in nature. For example previous studies from the cancer field have shown that histone hyperacetylation induced by HDACIs induced a pro‐apoptotic response in various cell types (Jeong *et al*. 2011; Bolden *et al*. 2013; Fujiki *et al*. 2013). Additionally, extensive microglial activation has been previously observed in this animal model of PD (Ahn and Jeon 2006; Pienaar *et al*. 2015), and there is evidence to suggest that HDAC activation may indeed potentiate such activation by increasing the expression of pro‐inflammatory mediators (Suuronen *et al*. 2003). Furthermore, it is important to remember that the profile of gene regulation and expression quantified here is in the frontal brain of the lactacystin‐lesioned brain, and hence the profile of gene expression changes in degenerating dopaminergic neurons of the SNpc themselves, may present a quite different picture. Further work should hence aim to provide a more thorough profile of gene expression changes, pro‐death and inflammatory factors included, using techniques such as microarray of extracts from the degenerating SNpc, in order to better understand the mechanism through which nicotinamide exacerbates dopaminergic neurodegeneration in this model.

Being a precursor for NAD+, nicotinamide is known to non‐selectively inhibit class III HDACs, inhibiting sirtuin1‐7 through competition binding to the NAD+ binding site of the sirtuin HDACs (Avalos *et al*. 2005). There is currently debate within the literature, however, as to the effects of these sirtuins in neuronal survival and neurodegeneration. For example, sirtuin1 and 5 are known to act neuroprotectively (Pfister *et al*. 2008; Dobbin *et al*. 2013; Donmez and Outeiro 2013), whereas sirtuin2, 3 and 6 are known to be neurotoxic (Pfister *et al*. 2008). Of the sirtuins, most research conducted in neurodegeneration has centred around sirtuin1 and 2: activation of sirtuin1 and inhibition of sirtuin2 emerging as novel targets for neuroprotection (Donmez and Outeiro 2013). Sirtuin1 is known to bind to a number of transcription factors (Donmez 2012), for example NF‐κB, p65, retinoic acid receptor β, forkhead box (FOXO) family of transcription factors, and most notable to neurodegeneration, peroxisome proliferator‐activated receptor gamma coactivator 1α which has long held therapeutic potential in PD (Zheng *et al*. 2010). Sirtuin1 activation has subsequently been shown to reduce αSyn aggregation through its up‐regulation of molecular chaperones (Donmez *et al*. 2012) and activation, via its deacetylation, of peroxisome proliferator‐activated receptor gamma coactivator 1α maintaining mitochondrial number and function (Austin and St‐Pierre 2012). Furthermore, sirtuin1 has recently been implicated in oxidative stress (Singh *et al*. 2017). Upon inhibition of sirtuin1 then, increased αSyn accumulation, in part by lack of molecular chaperones, mitochondrial DNA depletion and stress, and oxidative stress, are a likely consequence. Accordingly, sirtuin1 expression has been noted to be reduced in PD patients (Singh *et al*. 2017). Like sirtuin1, sirtuin2 also interacts with the FOXO family. More specifically sirtuin2 deacetylates FOXO3a which causes up‐regulation of Bim and subsequently induces caspase‐3 activated apoptotic cell death (Liu *et al*. 2012; erratum in Liu *et al*. 2013). Additionally, sirtuin2 is known to regulate αSyn inclusion number, size and cytotoxicity rescuing αSyn toxicity *in vivo* (Outeiro *et al*. 2007). Upon inhibition then, sirtuin2 is therefore thought to reduce αSyn toxicity and apoptotic cell death. Although nicotinamide is known to non‐selectively inhibit the sirtuin class of HDACIs, its IC_50_ values for each of the sirtuins vary greatly. Most notably, nicotinamide's IC_50_ for sirtuin1 is 85.1 μM whereas its IC_50_ for sirtuin2 is significantly less, at just 1.1 μM (Peck *et al*. 2010). This may therefore explain the toxic effects of nicotinamide observed in this study, that is lower doses of nicotinamide may be sufficient to inhibit sirtuin2 and yet also to a lesser degree sirtuin1. Whereas higher doses may be sufficient enough to induce extensive inhibition of sirtuin1 hence the dose‐dependent toxic effects of this drug in this study. Given the nuclear localization of sirtuin 1 too, and its effects on transcription factor activity (Lee and Goldberg 2013), such a hypothesis would also be consistent with the over‐expression profile observed in many genes upon treatment of animals with the high dose of nicotinamide. Data presented here therefore highlight the importance of target specificity within this class of HDACs, given the contrasting effects of its individual isoforms, and supports the much needed development of specific class III HDACIs.

## Conclusions

By using a clinically relevant drug testing platform, here we demonstrate that nicotinamide's inhibition of the sirtuin HDACs (class III) is dose‐dependently neurotoxic in the lactacystin rat model of PD, when nicotinamide administration begins 7 days post‐lesion of the SNpc, where behavioural symptoms and MRI changes, dopaminergic cell loss and molecular hallmarks of neurodegeneration in the animal model are already observed. The toxic effects of nicotinamide's sirtuin inhibition are associated with exacerbation of behavioural motor‐based symptoms, the neuropathological progression of the model as detected through MRI, and nigral dopaminergic neurodegeneration. These changes were accompanied by reversal of lactacystin‐induced histone hypoacetylation resulting in histone hyperacetylation and an up‐regulation of neuroprotective and neurotrophic factors. Such up‐regulations, however, did not translate to neuroprotection, rather exacerbated neurodegeneration suggestive that the histone hyperacetylation observed may have also increased expression of neurotoxic factors not studied here. These findings therefore highlight the importance of target specificity within this class of HDACs and demonstrate the contrasting effects of sirtuin inhibition upon cell survival in this, compared to other animal models of PD.

## Acknowledgments and conflict of interest disclosure

IFH was supported by a UK Medical Research Council PhD Studentship. The authors would also like to acknowledge and thank Prof Sebastien Ourselin, for his supervision of NMP. The authors declare that they have no competing interests.

All experiments were conducted in compliance with the ARRIVE guidelines.

## Author contributions

IFH contributed to the design of the study, performed all experiments and analysis, and wrote the manuscript. NP performed the tensor‐based morphometry analysis, and contributed to the manuscript critique. DTD conceived and helped design the study, and contributed to the manuscript critique.

## Supporting information


**Figure S1.** Cellular and Molecular Effects of Nicotinamide Treatment in Healthy Control Rats.
