# Supplementary material for: The histone deacetylase inhibitor nicotinamide exacerbates neurodegeneration in the lactacystin rat model of Parkinson's disease
Source: J Neurochem. 2018 Nov 26;148(1):136–56. doi: 10.1111/jnc.14599 (PMC6487684; doi:10.1111/jnc.14599)

The Histone Deacetylase Inhibitor Nicotinamide Exacerbates Neurodegeneration in the Lactacystin Rat Model of Parkinson's Disease

Ian F. Harrison[1,2]*, **Nicholas M. Powell**[1,3], and **David T. Dexter**[2]

(1) UCL Centre for Advanced Biomedical Imaging, Division of Medicine, University College London, London, WC1E 6DD, UK

(2) Parkinson's Disease Research Group, Centre for Neuroinflammation and Neurodegeneration, Division of Brain Sciences, Department of Medicine, Imperial College London, London, W12 0NN, UK

(3) Translational Imaging Group, Centre for Medical Image Computing, University College London, London, WC1E 6BT, UK

* Corresponding author: Dr Ian F Harrison
Centre for Advanced Biomedical Imaging
Division of Medicine
University College London
WC1E 6DD, UK
Tel.: +44 (0)20 7679 6448
Email: ian.harrison@ucl.ac.uk

Supplementary Figure 1 – Cellular and Molecular Effects of Nicotinamide Treatment in Healthy Control Rats

In addition to the four treatment groups detailed in figure 1, a separate cohort of male Sprague-Dawley rats (250±10g, n=7) were injected daily for 28 days with the higher dose of nicotinamide used in the study (500mg/kg i.p.), to determine the effects of nicotinamide alone on the cellular and molecular readouts reported in the four study treatment groups. After the final i.p. injection on day 28, these animals were sacrificed and brain tissue harvested for subsequent analysis. For the purposes of comparison in this figure, data from this group (Lacta(-)NTA(++)) is compared to data from the surgically naïve group of animals similarly treated with saline for 28 days (Lacta(-)NTA(-)) reported in the main study figures. Representative examples of the TH and Nissl stained left (Ai and Aiii) and right (Aii and Aiv) SNpc of rats treated with either saline (Ai and Aii) or nicotinamide (Aiii and Aiv). Scale bar equal to 500µm. Stereologically estimated (B) TH+ and (C) Nissl+ neuron numbers in the SNpc of rats suggest that nicotinamide treatment alone does not have any effect on nigral dopaminergic or neuronal cell numbers. Systemic injection of nicotinamide however induces subtle bilateral increases in histone acetylation (AcH3-Lys9) in the brain. (D) Densitometry analysis of the AcH3-Lys9 band relative to the β-actin band used as a loading control. (E) Representative blot of data presented in (D). (F) qRT-PCR results demonstrate subtle changes in expression levels of numerous genes as a result of nicotinamide treatment. n = 7 per group. Abbreviations: L, left; R, right.

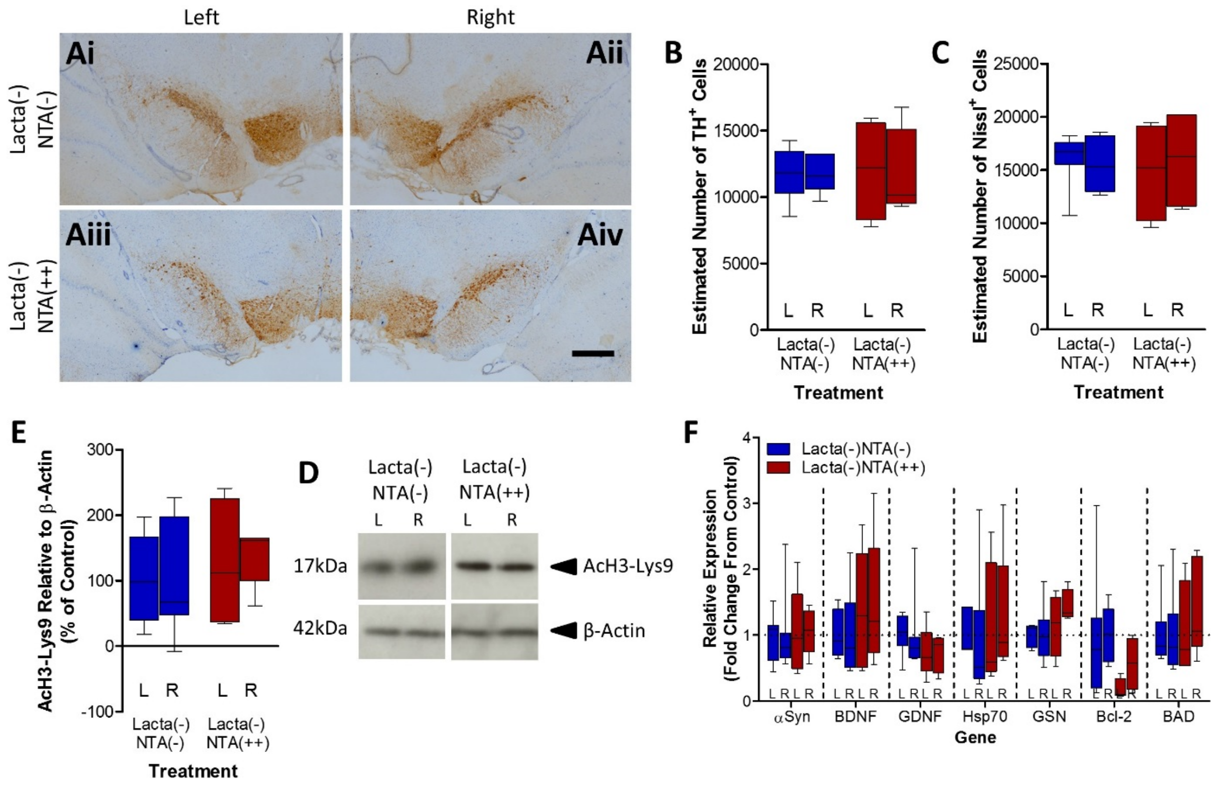

Supplement: Supplementary file 1 — Figure S1. Cellular and Molecular Effects of Nicotinamide Treatment in Healthy Control Rats. [file JNC-148-136-s001.pdf]
